# Stereo cell: A new approach to the next generation of clinical precision medicine

**DOI:** 10.1002/ctm2.70537

**Published:** 2025-11-28

**Authors:** Wanxin Duan, Mingjie Wang, Yifei Liu, Celine Desoyer, Christian Baumgartner, Xiangdong Wang

**Affiliations:** ^1^ Department of Pulmonary and Critical Care Medicine Zhongshan Hospital Fudan University Shanghai Medical College Shanghai China; ^2^ Shanghai Institute of Clinical Bioinformatics Shanghai China; ^3^ Fudan University Center of Clinical Bioinformatics Shanghai China; ^4^ Department of Gastroenterology Ruijin Hospital of Shanghai Jiao Tong University Shanghai China; ^5^ European Stereo Cell Center (ESCC) Institute of Health Care Engineering with European Testing Center of Medical Devices Graz University of Technology Graz Austria; ^6^ Department of Computer Science and Biomedical Engineering Institute of Health Care Engineering with European Testing Center of Medical Devices Graz University of Technology Graz Austria

**Keywords:** precision medicine, sc‐surfaceome, single‐cell multi‐omics, spatiotemporal, stereological cell biomedicine

## Abstract

Precision medicine has evolved through distinct phases, from the origins of the Human Genome Project to mutation‐based targeted therapies. This editorial posits that “stereological cell biomedicine” could be a new approach promoting the development of the next generation of precision medicine. This emerging discipline transitions the focus from genomic data to the multi‐dimensional and spatiotemporal complexity of single cells. Driven by advances in Stereo single‐cell multi‐omics (Stereo Cell‐seq), spatial transcriptomics (Stereo‐seq), and single‐cell surfaceomics (sc‐surfaceome), this approach aims to capture the stereologically dynamic interactions between organelles within a cell and between cells in the tissue. We argue that understanding the spatiotemporal location of molecules, particularly protein interactions at organelle interfaces and on the cell surface, is as critical as their abundance for defining cellular function in health and disease. Integrating these high‐resolution measurements with artificial intelligence and computational modelling will bridge the gap between advanced omics and pathology. Initiatives such as the newly established European Stereo Cell Center (ESCC) signal a global shift towards this new paradigm, which promises to unlock novel diagnostic biomarkers and therapeutic targets for truly multi‐factorial and dynamic precision medicine.

Precision medicine has been developing for decades and has become a well‐known and accepted concept and clinical practice and was initiated on the basis of the International Human Genome Project. This project aimed to sequence approximately 3 billion nucleotides on DNA and generate the human genome sequence map as the first phase of knowledge enrichment, starting in 1990.^1^ During this stage, DNA sequencing technologies developed rapidly, high‐quality genome data were generated and analyzed, and genomic variations associated with diseases were recognized and proposed for diagnosis and therapy.^2^ Since “Towards Precision Medicine” was proposed in 2011,^3^ a large number of corresponding publications have been published, emphasizing the importance of precision medicine for improving disease outcomes. A national program, called the “Precision Medicine Initiative”, was launched to promote personalized genomics research and individualized medicine for diseases in January of 2015, as the second phase of concept discovery. During this phase, knowledge of and proposals for precision medicine were extended to various diseases, and the number of precision medicine centres increased worldwide. For instance, Fudan University Zhongshan Hospital established a Center of Clinical Precision Medicine in May 2015 and a clinical multidisciplinary team in December 2018. As one of the earliest clinical organizations for precision medicine, it explores the development of mutation‐based target therapies. The multidisciplinary therapy strategy of precision medicine was implemented in clinical practice, to dynamically monitor clinical phenomes and gene mutations of patients with multiple metastases during the treatment.^4^ More and more targeted diagnostics, therapies, and strategies were developed as the clinical practice of precision medicine.^5^ For example, the constitutively activated tyrosine kinase (BCR‐ABL) was selected as a molecular target in chronic myeloid leukemia and inhibited by specifically designed drugs.^6^ This marked a milestone in the third phase of precision medicine entering clinical practice, even though the target had been recognized a decade earlier. Precision medicine is entering a new era of multi‐dimensional, multi‐layered, multi‐oriented, and multi‐factorial spatiotemporal diagnoses and therapies, empowered by gene editing, molecular imaging, biobanks, multi‐omics, and immunotherapy.^7^, ^8^, ^9^

Recently, single‐cell measurements were suggested as one of the routine measurements in clinical haematology. These measurements provide comprehensive profiles of circulating immune cells and their subtypes/subsets based on differentially expressed genes. This is a critical initiative in clinical and translational medicine.^10^, ^11^, ^12^, ^13^, ^14^ Haematological and immunological staining can detect about 5–20 subtypes of each immune cell in the circulation on the basis of cell morphology and protein markers. In contrast, single‐cell RNA sequencing (scRNA‐seq) can detect about 20–100 subtypes and about 1000 subsets defined by cell identity marker gene panels (ciMGPs; Figure 1A). The number of clinical trials using scRNA‐seq and multi‐omics for disease‐specific biomarker validation or monitoring drug efficacy is increasing.^15^ Although many challenges must be overcome, single‐cell biology can be an important part of precision medicine and has great potential for market development.^15^, ^16^ One of these challenges is providing solid evidence to prove the accuracy and specificity of ciMGPs for each subtype/subset (Figure 1A). This is because the sources and discoveries of cell subset‐specific ciMGPs have hardly been fully validated, some of genes are repeated in various cell subsets, and the same subset is marked with different ciMGPs.^17^, ^18^ With the rapid development of scRNA‐seq, a large number of cell types and subtypes are being identified using a combination of cell morphology and protein biomarkers followed by scRNA‐seq, a technique coined “Stereo‐cell” technology.^19^ In clinical hematology, morphological images of cells are used to confirm the cell types. Cell‐specific surface proteins, identified using monoclonal antibodies, are used to define cell subtypes as one of the standardized practices. Integrating identities between proteins and ciMGPs may be suitable for identifying subsets of each subtype (Figure 1B), improving the accuracy and specificity of cell subtypes/subsets for clinical diagnosis and monitoring. Spatial transcriptomics, as part of molecular pathology, provides tissue images, cell morphology, single‐cell transcriptomic profiles, and cell–cell interactions. Dynamic images of these can be visualized for stereological cell phenomes (Figure 1C). These images represent the existing and real tissue structure and pathology using Stereo‐seq, even though some cell and tissue information can be lost due to the section per se. Meanwhile, the dynamic remodelling of the tumour microenvironment and the molecular regulations of the tissue remodelling can be illustrated using a stereo‐cell (Figure 1D).

**FIGURE 1:**
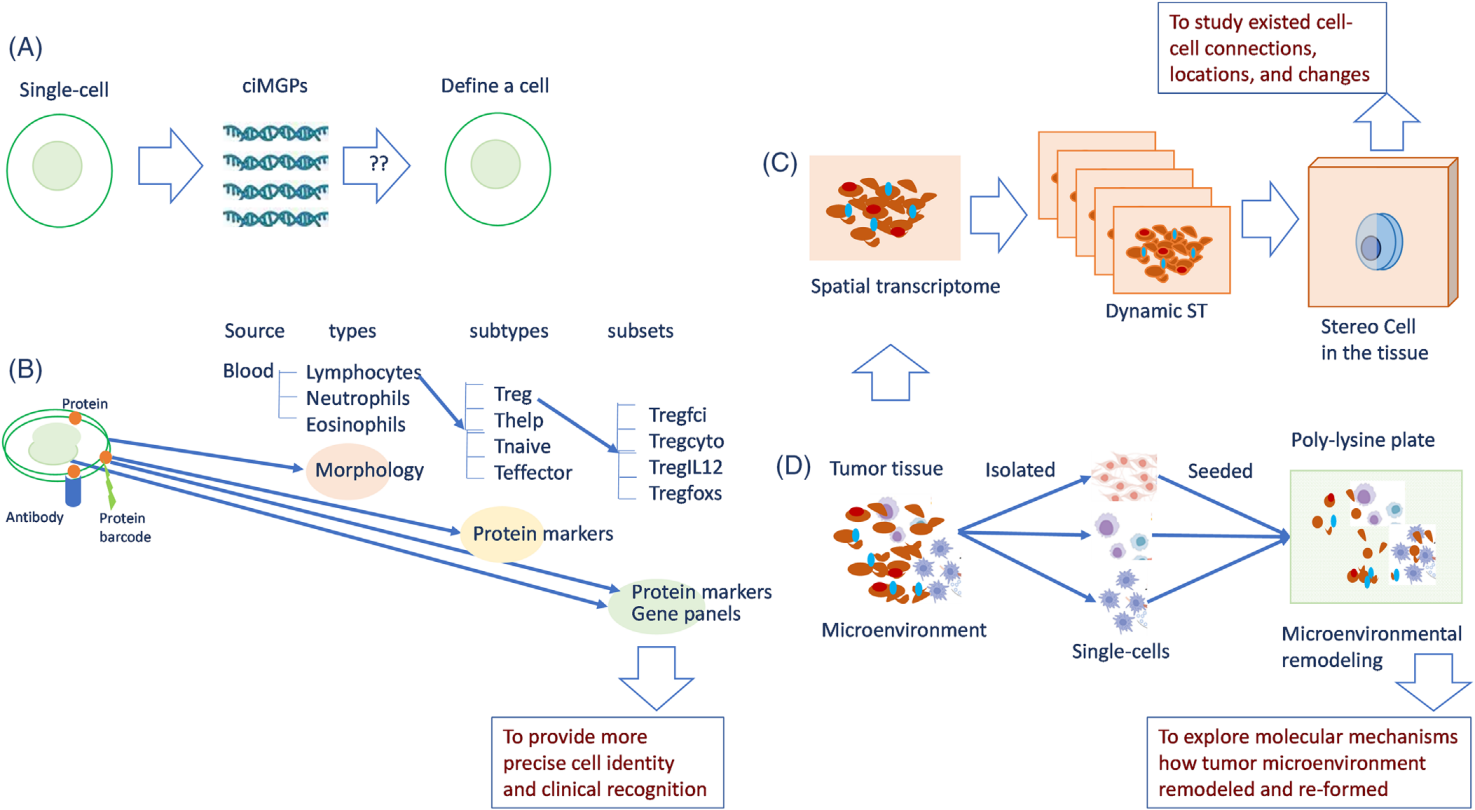
Conceptual framework for precise cell identification and spatiotemporal analysis in stereological cell biomedicine. This figure illustrates the integrated, multi‐modal strategies required for the next generation of precision medicine, moving beyond mere gene expression to capture the morphological, protein, and spatiotemporal context of single cells. (A) Challenges in defining cell identity via gene panels alone. This panel highlights the foundational method of using cell identity marker gene panels (ciMGPs) derived from single‐cell RNA sequencing (scRNA‐seq) to define a cell. The question marks emphasize the challenges and uncertainties involved in fully and accurately defining complex cell identities based solely on a limited set of transcriptional markers, as discussed in the text. (B) Multi‐modal strategy for precise cell identity and clinical recognition. This panel proposes an integrated approach to enhance the accuracy and specificity of cell identification. It combines: (1) Source and morphology (Morpho): Traditional haematological classification based on cell source (e.g., blood) and morphological characteristics to define major cell types (e.g., lymphocytes). (2) Protein markers: Cell‐specific surface proteins, identified using monoclonal antibodies, are utilized to define precise subtypes (e.g., Treg, Thelp). (3) Integration of protein markers and gene panels: The confluence of surface protein profiles and scRNA‐seq‐derived gene panels is used to robustly identify highly specific subsets (e.g., Tregcl1, Tregcl2). This combinatorial strategy is crucial to provide more precise cell identity for clinical recognition and diagnosis. (C) Visualization of the stereo cell in situ using spatial transcriptomics. This panel conceptualizes the Stereo Cell by integrating spatial omics with structural imaging. Spatially resolved transcriptomics (e.g., Stereo‐seq) provides multi‐layered data from sequential tissue sections, analogous to a dynamic CT. This process allows for the computational or physical reconstruction of the Stereo Cell in the tissue, enabling the study of established cell‐cell connections, positional coordinates, and molecular changes in their native tissue context. (D) Dynamic modelling of microenvironmental remodelling ex vivo. This panel depicts an experimental workflow designed to explore the molecular mechanisms of tissue dynamics. Tumour tissue and its microenvironmental components are harvested and dissociated into individual single cells and corresponding Habitat (extracellular matrix/stromal elements). These components are then re‐seeded onto a compatible substrate (e.g., polylysine plate) for co‐culture. This model facilitates the observation and mechanistic study of microenvironmental remodelling and reformation, allowing researchers to explore how cellular components and molecular regulations dynamically interact outside the body.

Stereological cell biomedicine is a new discipline combining single‐cell‐based stereology, biology, and medicine. It provides multi‐dimensional images of a cell, as well as information on spatiotemporal interactions and functions among intracellular organelles, and the comprehensive molecular regulations occurring at intracellular locations.^20^ The protein content per cell is approximately 10 billion (10^10^) and varies depending on the cell type, function, location, and disease in humans. Of these proteins, about 10^6^ are in the cell surfaceome, and the areal density is approximately 20 000–40 000 membrane proteins per µm^2^ of cell surface.^21^ In addition to the quantity and density of proteins, their spatiotemporal locations of proteins on the single‐cell surface (sc‐surfaceome) play an important role in intercellular interactions and communications, and are highly specific to disease. The rapid development of surfaceome measurements is providing new insights into the complexity, characteristics, and function of sc‐surfaceomes, offering a deeper understanding that will inform the identification of diagnostic biomarkers and therapeutic targets for the next generation of precision medicine. sc‐surfaceomics will demonstrate the profiles and patterns of protein–protein interactions and membrane protein complexes on the cell surface, defining extracellular post‐translational modifications, for example, cell surface glycosylation, proteolytic remodelling, and the secretome.^22^ A recent milestone study on the spatiotemporal distribution and interaction of human proteins at the subcellular level demonstrated that intra‐/inter‐organelle protein communication and regulation depend heavily on dynamic spatial alterations, rather than protein abundance.^23^ The “Hein” strategy uses organelle immunocapture coupled to mass spectrometry to define the subcellular localization of over 7600 proteins, spatial networks, and interconnections between cellular compartments (with or without the membranes), to understand the molecular mechanisms by which the organelles interact and are regulated. This approach is important for investigating the nature and qualities of inter‐organellar communications within a stereo cell, as it involves measuring connectivity densities and protein spatial networks at organelle interfaces. The spatiotemporal interactions between organelles will determine and control cell function, generating a large number of disease‐specific targets and leading to new generations of precision medicine.

Supported by stereological computational and systems approaches, combining artificial intelligence, computational modelling, digital proteomics, and spatiotemporal multi‐omics, stereological cell biomedicine will develop much faster. This integrated computational framework can be utilized to reconstruct, simulate, and predict stereo cell structures and interactions in health and disease. It has been suggested that it can bridge the gap between advanced omics and traditional pathology, providing a comprehensive profile of rare detectable cells, and enabling clinically interpretable diagnoses.^24^ The impact and value of stereological cell biomedicine in the next generation of precision medicine will grow tremendously as our understanding of stereo single‐cell complexity increases and spatiotemporal measurements and AI‐driven computational modelling develop rapidly. The European Stereo Cell Center (ESCC) at Graz University of Technology (TU Graz), Austria, was established in October 2025 (https://stereo‐cell.org/), as a dynamic platform for stereological cell biomedicine promoting collaboration, innovation and education, and connecting researchers, clinicians and engineers from various disciplines and continents. As a global initiative in stereological cell biomedicine, we anticipate that more centres and institutes will emerge, accelerating discoveries and contributing to the next generation of precision medicine based on stereological cell biomedicine.

## AUTHOR CONTRIBUTIONS

Wanxin Duan, Mingjie Wang, Yifei Liu, and Celine Desoyer contributed to the preparation of the article, scientific discussion, and writing. Christian Baumgartner and Xiangdong Wang were responsible for the idea generation and scientific design as well as writing.
